# Impact of tuberculosis on mortality among HIV-infected patients receiving antiretroviral therapy in Uganda: a prospective cohort analysis

**DOI:** 10.1186/1742-6405-10-19

**Published:** 2013-07-13

**Authors:** Rong Chu, Edward J Mills, Joseph Beyene, Eleanor Pullenayegum, Celestin Bakanda, Jean B Nachega, P J Devereaux, Lehana Thabane

**Affiliations:** 1 Department of Clinical Epidemiology and Biostatistics, McMaster University, Hamilton, Canada; 2 Biostatistics Unit, 3rd Floor Martha, Room H325, St Joseph’s Healthcare Hamilton, 50 Charlton Avenue East, Hamilton, ON L8N 4A6, Canada; 3 Faculty of Health Sciences, University of Ottawa, Ottawa, Canada; 4 The AIDS Support Organization (TASO), Kampala, Uganda; 5 Department of Medicine and Centre for Infectious Diseases, Stellenbosch University, Cape Town, South Africa; 6 Departments of Epidemiology and International Health, Johns Hopkins Bloomberg School of Public Health, Baltimore, MD, USA; 7 Hamilton Health Sciences, Population Health Research Institute, Hamilton, Canada

**Keywords:** Antiretroviral therapy, HIV, Tuberculosis, Propensity score methods, Uganda, Prospective cohort study

## Abstract

**Background:**

Tuberculosis (TB) disease affects survival among HIV co-infected patients on antiretroviral therapy (ART). Yet, the magnitude of TB disease on mortality is poorly understood.

**Methods:**

Using a prospective cohort of 22,477 adult patients who initiated ART between August 2000 and June 2009 in Uganda, we assessed the effect of active pulmonary TB disease at the initiation of ART on all-cause mortality using a Cox proportional hazards model. Propensity score (PS) matching was used to control for potential confounding. Stratification and covariate adjustment for PS and not PS-based multivariable Cox models were also performed.

**Results:**

A total of 1,609 (7.52%) patients had active pulmonary TB at the start of ART. TB patients had higher proportions of being male, suffering from AIDS-defining illnesses, having World Health Organization (WHO) disease stage III or IV, and having lower CD4 cell counts at baseline (p < 0.001). The percentages of death during follow-up were 10.47% and 6.38% for patients with and without TB, respectively. The hazard ratio (HR) for mortality comparing TB to non-TB patients using 1,686 PS-matched pairs was 1.37 (95% confidence interval [CI]: 1.08 – 1.75), less marked than the crude estimate (HR = 1.74, 95% CI: 1.49 – 2.04). The other PS-based methods and not PS-based multivariable Cox model produced similar results.

**Conclusions:**

After controlling for important confounding variables, HIV patients who had TB at the initiation of ART in Uganda had an approximate 37% increased hazard of overall mortality relative to non-TB patients.

## Background

The total number of people living with human immunodeficiency virus (HIV) reached 34.0 million (31.6 – 35.2 million) worldwide by the end of 2010, [1] with the majority in Sub-Saharan Africa. One-third of HIV-infected people are estimated to be co-infected with *Mycobacterium tuberculosis* (TB) which can activate or reactivate during the initiation of antiretroviral therapy (ART) due to immune reconstitution inflammatory syndrome (IRIS). However, TB incidence rates vary according to geography and patients’ degrees of immunosuppression. The incidence proportion of active TB in HIV-infected patients with latent TB infection is about 10% per year compared to 10% per lifetime for an HIV-uninfected individual [2]. TB is a leading cause of HIV-related death [3]. Recent trial data have shown that early initiation of ART (within two weeks) during TB therapy can improve survival for patients with co-infection [4-6]. Guidelines and policies on joint HIV/TB interventions have been developed to promote synergies between TB and HIV/AIDS prevention and care activities, [7-9] aimed at reducing morbidity and mortality in co-infected patients. On the other hand, joint treatment containing ART and anti-TB drugs may be complicated by overlapping toxicity profiles, complex drug-drug interactions, and IRIS [10-13].

To date, the association between active TB and mortality in HIV-infected patients who receive ART is poorly understood, particularly in settings with a relatively lower TB prevalence, such as Eastern Africa [9,13,14]. An observational study from Tororo, Uganda, comprising 1,044 HIV patients showed that TB at the initiation or during follow-up of ART was associated with a 4.7-fold increase in cumulative mortality relative to those without TB [15]. Nevertheless the association of TB disease and mortality was clearly confounded by degree of immunosuppression [15,16]. Several studies in more developed countries suggest incident TB substantially increases the risk of AIDS-related mortality among HIV patients receiving ART [17,18]. A recent meta-analysis suggested little impact of TB on mortality in HIV patients receiving ART [19]. Small numbers of co-infected patients, different procedures of baseline confounding adjustment, and incomplete information on clinical outcomes have led to uncertainty of the impact of active TB on subsequent mortality among co-infected patients on ART and the optimal timing of treatment of both diseases.

Our study involved a large prospective cohort of HIV-infected adult patients in Uganda, and had two objectives: (1) to assess the effect of active pulmonary TB disease at ART initiation on overall survival among HIV patients during ART treatment by adjusting for potential confounders using propensity score (PS) methods, [20-22] and (2) to explore the robustness of the study findings by comparing results from three PS methods (matching on PS, stratifying on PS, adjusting for PS as regression covariate) to conventional (not PS-based) multivariable Cox regression model. These results may help accurately plan TB and HIV/AIDS management activities in HIV patients with TB co-infection.

## Methods

### Setting, participants and data collection

Our prospective cohort contained HIV-infected patients initiating ART at ten service centres managed by the AIDS Support Organization (TASO) across different settings in Uganda since 2000. Patients received ART from experienced medical staff at TASO clinics, outreach clinics in rural areas, and through community-based treatment programs. Details of the treatment program have been published previously [23]. The standard regimen was two nucleoside reverse transciptase inhibitors and one non-nucleoside reverse transcriptase inhibitor. The current study included adult patients (aged 14 years or older at ART initiation) enrolled between August 2000 and June 2009 (inclusive) as part of an ongoing observational study intended to evaluate the programmatic delivery of services. After ART initiation, patients were scheduled for clinic visits at least every three months. Patients’ demographic, clinical, psychosocial, and medication use data were collected by clinicians, and field workers using standardized forms at ART initiation, and each visit.

At the time of data collection, national guidelines in Uganda used three strategies for initiating treatment of TB in a co-infected patient: (1) patient with TB and CD4 cell count < 250 cells/mm^3^ or a patient with extra-pulmonary TB or WHO stage IV disease: start TB therapy first and when tolerated (usually within two to six weeks) then introduce ART; (2) pulmonary TB and CD4 250–350 cells/mm^3^: start TB therapy for two months then introduce ART, and; (3) pulmonary TB and CD4 > 350 cells/mm^3^: defer ART, monitor clinically and also do CD4 cell counts regularly; re-evaluate the patient at eight weeks and the end of TB treatment [24].

### Primary outcome, exposure and potential confounding variables

Our primary outcome was time from the initiation of ART to death for any reason after the initiation of ART between August 2000 and June 2009. The primary exposure was active pulmonary TB as evidenced by sputum smear-positive results at the initiation of ART, or suspected TB regardless of sputum results followed up by radiography. Fourteen variables containing clinical and demographic characteristics and medicine history measured at the beginning of ART (baseline), were considered in the study and their roles as potential confounders were investigated. The 14 baseline covariates were gender, age, CD4 count, World Health Organization (WHO) clinical disease stage of HIV/AIDS, presence of AIDS-defining illness, TASO service centre, calendar year of ART initiation, education, marital status, partner sero-status, sexual activity, sexually transmitted infection, history of pneumocystis pneumonia (PCP), and toxoplasmosis. A potential confounder was defined here as a baseline covariate that was likely to associate with the overall survival and baseline disease status of TB. The assessment of association between the covariates and the outcome was primarily based on published literature [25-29]. We also hypothesized the associations between overall survival and some covariates that pertained to the study setting. For example, the variation in political and socioeconomic factors and unmet demand for healthcare workers across the 10 Ugandan study sites were likely to result in difference in patient outcomes [30]. Patient sexual dynamics could potentially affect prevention and treatment for HIV/AIDS in African countries [31-34]. Literature suggested that people with weak immune systems were generally more likely to have active TB disease [35]; and among HIV patients, male gender, low CD4 count, high viral load, severe disease stage, and low socioeconomic status were possible risk factors of active TB disease. We carefully examined the distributions of the 14 covariates between TB and non-TB groups at baseline (see “Statistical analysis” section). We analyzed age and CD4 count as continuous variables, and the other 12 covariates as categorical data coded by a reference level and dummy indicators (Additional file 1).

### Statistical analysis

We adopted PS methods to control for confounding, and estimated the causal effect of TB at the initiation of ART on overall survival of HIV patients since their initiation of ART using Cox regression models. We reported the crude and adjusted effects of TB using hazard ratios [HRs]. A patient was censored at the time they were lost to follow-up, or at the end of the study if still alive. PS methods have been increasingly used to control for baseline confounding in observational studies [20-22,36-40]. PS methods are more advantageous than adjusting for multiple covariates simultaneously in a single outcome model when numerous confounders need to be accounted for and the outcome is rare. PS methods provide an easy means to compare distributions of confounding variables between exposure groups within PS-stratified sets. It can be difficult to examine balancing distributions of confounders post-adjustment using the conventional multiple regression methods. We chose Cox regression on PS-matched pairs as the primary method of analysis because empirical evidence suggested PS-matching provides better control for confounding than other PS methods, [41,42] and statistical diagnostic tools for assessing the balance of confounders between PS-matched pairs were readily available [40,43,44]. Specifically, we fit a Cox proportional hazards (PH) model in a dataset containing 1,686 TB cases and 1,686 PS-matched non-TB patients to control for observed potential confounding using a two-step procedure. First, we modeled the relationship between having active TB at baseline and the observed baseline covariates using a logistic regression model (“PS model”). We employed an iterative approach [45] to build the PS model by starting with the 14 observed baseline covariates; we then considered pairwise interactions, and polynomial terms of the continuous variables (age and CD4 cell count) in the PS model. We ultimately included such terms in the model if adding them improved the predictive power of the PS model and its ability to balance covariates between PS-matched pairs. Greater predictive power of the PS model (i.e. how well covariates included in the PS model predict baseline TB disease status) was likely to result in greater balance; the performance of matching was also affected by other factors such as the matching algorithm and associated tuning parameters (e.g. caliper width). We aimed to match each active TB patient to one non-TB patient with the closest logit of the estimated PS, within a caliper of 0.2 of the standard deviation of the logit of the estimated PS [46]. This matching method was shown to lead to optimal estimation of exposure effects in various simulation settings [41,47]. Propensity score is a balancing score, so we assessed PS model adequacy (selection of variables and functional form) based on examining the similarity of the joint distribution of multiple baseline covariates between TB and non-TB patients with the same estimated propensity score [42]. Balance diagnostics of baseline covariates consisted of a series of numerical and graphical measures, including standardized difference, and for continuous covariates, ratio of variance, five-number summaries, quantile-quantile plot, nonparametric density plot, empirical cumulative distribution function and side-by-side boxplot. We repeated the PS modeling – balance diagnostics steps until we were satisfied with the resulting balancing distribution. Second, we fit a stratified Cox PH model to estimate the impact of baseline TB on survival. As outcomes within PS-matched pairs are not independent, we stratified on pairs to controls for within-pair homogeneity, allowing baseline hazard functions to vary across matched strata [45,48]. The appropriateness of the PH assumption was assessed using log-log survival curves.

We applied two other PS methods for confounding control as supportive secondary analyses to assess the robustness of the results of the primary analysis, namely, stratified Cox regression on quintiles of the estimated PS, and a Cox model adjusting for PS and its quadratic and cubic terms as covariates. Conventional (not PS-based) Cox regression directly modeling the effect of TB on overall survival, adjusting for multiple baseline covariates in the same form as included in the final PS model, was also performed.

Complete information was recorded for the study sample on TB status at ART initiation and on all but four baseline covariates, namely, age (8% missing), CD4 count (17.3% missing), sexual activity (17.3% missing) and WHO stage of HIV/AIDS (34.4% missing). Excluding patients with missing data on any of the covariates would have led to a loss of 48% of subjects and 86% of death events. Such exclusions could have affected the stability of statistical models (PS-based or not) due to low event rate and jeopardized the internal validity and generalizability of the study. Therefore, we performed all analyses on five multiple imputation (MI) datasets [49]. We used Markov chain Monte Carlo method assuming multivariate normality to create a monotone missing pattern, followed by separate imputations for the continuous (age, log CD4 count) and categorical (sexual activity and stage of disease) variables using linear and logistic regression models. A number of baseline characteristics (TB status, gender, TASO sites, AIDS status, education level, marital status, partner sero-status, sexually transmitted infection, calendar year of ART initiation, and history of PCP or toxoplasmosis) and follow-up variables (death, regimen switch, patient adherence, and AIDS status post-ART initiation) were included in the imputation procedure. The follow-up variables were assessed after the initiation of ART and before the end of the study (death, lost to follow-up or June 2009, whichever happened first). Patient adherence was assessed at two weeks, two months and thereafter at six months after ART initiation, based on a composite of pharmacy monitored drug possession ratio, pharmacy refill records, and a three day recall report by patients or care givers. TASO calculated the mean adherence to ART for each patient. Adequate clinical adherence was defined as ≥95% (i.e. adherence = yes; Additional file 1) [50,51]. No post-ART variables were included in PS or Cox regression models as they could be causal intermediates between baseline TB status and death, and accounting for them in the models could induce bias. While the same covariates were used to achieve prognostic balance on the five imputed datasets, propensity scores and baseline TB – mortality association were estimated separately for each individual dataset. We then calculated the overall estimate of TB effect, using Rubin’s rule [49].

We conducted statistical analyses in SAS version 9.2 (Cary, NC), R 2.14.0 (R Core Development Team) and Stata 10 (College Station, TX).

### Ethics approval

University of British Columbia, University of Ottawa, and Mbale Regional Referral Hospital research ethics boards approved this study.

## Results

### Participant baseline characteristics

The study cohort consisted of 22,477 HIV-infected adult patients aged 14 years or older who started ART between August 2000 and June 2009 (Additional file 2: Figure S1). A total of 1,690 of 22,477 patients (7.52%) had active pulmonary TB disease at the initiation of ART. Table 1 indicates TB patients had lower CD4 counts (p < 0.001) and more advanced clinical stages (p < 0.001) at the start of ART. TB patients tended to be younger, had higher proportions of male, AIDS-defining illnesses, and a history of PCP (p < 0.001). Higher proportions of the TB patients were managed by the Entebbe, Jinja, Tororo and Mulago regional centres, whereas higher proportions of the non-TB patients were observed in Masaka, Mbale, Mbarara, Masindi, and Soroti regions. Summary statistics of the four baseline covariates with missing values was similar in the original and imputed datasets (Additional file 3).

**Table 1:** **Comparison of baseline and follow**-**up characteristics between HIV**-**infected patients with and without co**-**infection of tuberculosis in the unmatched sample with imputed covariates** (**for a single multiple imputation dataset**)

**Characteristics**	**TB: yes**	**TB: no**	**P-value**	**Standardized difference**
	**(n = 1690)**	**(n = 20787)**		**(Ratio of variance for continuous covariate)**
**Baseline characteristics**				
***Demographics***				
Age (year)				
Mean (SD)	36.87 (8.78)	37.93 (9.48)	<0.0001	−0.117 (0.86)
Min	14	14		
25th percentile	31	31		
50th percentile	36	37		
75th percentile	42	43		
Max	73	93		
Male: n (%)	670 (39.64)	6216 (29.90)	<0.0001	0.206
***HIV severity***				
CD4 count				
Mean (SD)	143.39 (149.94)	170.82 (172.26)	<0.0001	−0.170 (0.76)
Min	0	0		
25th percentile	45	66		
50th percentile	111	139		
75th percentile	193	208		
Max	1701	1983		
WHO stage: n (%)				
1	43 (2.54)	992 (4.77)	<0.0001	-
2	441 (26.09)	11013 (52.98)		
3	999 (59.11)	7173 (34.51)		
4	207 (12.25)	1609 (7.74)		
AIDS: n (%)	493 (29.17)	3881 (18.67)	<0.0001	0.248
***History of drug use* &*illnesses *: *n *(%)**				
Pneumocystis pneumonia	28 (1.66)	101 (0.49)	<0.0001	0.114
Toxoplasmosis	11 (0.65)	101 (0.49)	0.3542	0.022
Sexually transmitted infection	341 (20.17)	4353 (20.94)	0.4578	−0.019
***Partner information*: *n *(%)**				
Married poly	125 (7.40)	1801 (8.66)	0.0734	−0.047
Partner sero-positive	417 (24.67)	5801 (27.91)	0.0043	−0.073
Sexually active	1224 (72.43)	15272 (73.47)	0.3508	−0.023
***Other characteristics*: *n *(%)**				
Site				
ENT	378 (22.37)	1950 (9.38)	<0.0001	-
GUL	161 (9.53)	1891 (9.10)		
JIN	276 (16.33)	2586 (12.44)		
MAS	130 (7.69)	2246 (10.80)		
MBL	144 (8.52)	2446 (11.77)		
MBR	116 (6.86)	2641 (12.71)		
MSD	61 (3.61)	1254 (6.03)		
MUL	211 (12.49)	2390 (11.50)		
SOR	29 (1.72)	1546 (7.44)		
TOR	184 (10.89)	1837 (8.84)		
Education (Higher institute)	58 (3.43)	623 (3.00)	0.3158	0.025
ART start year since 2000				
0	0 (0)	2 (0.01)	<0.0001	-
4	23 (1.36)	946 (4.55)		
5	453 (26.80)	4532 (21.80)		
6	373 (22.07)	3627 (17.45)		
6	507 (30.00)	6715 (32.30)		
8	330 (19.53)	4943 (23.78)		
9	4 (0.24)	22 (0.11)		
**Follow**-**up characteristics**				
Death: n (%)	177 (10.47)	1326 (6.38)	<0.0001	0.148
Median (IQR) length of follow-up (days)	627.5 (205–1034)	646 (306–1045)	-	-

Note: For continuous variables, mean and standard deviation for each group, standardized mean difference and ratio of variances between TB and no-TB patients are reported. For categorical variables, frequency and percentage of the specified level, and standardized difference between TB and no-TB patients are reported, unless noted otherwise. *MI* multiple imputation, *SD* standard deviation, *WHO* World Health Organization, *HIV* human immunodeficiency virus, *AIDS* acquired immune deficiency syndromes, *ENT* Entebbe, JIN: Jinja, *MAS* Masaka, *MBL* Mbale, *MBR* Mbarara, *MUL* Mulago, *TOR* Tororo, *GUL* Gulu, *SOR* Soroti, *MSD* Masindi, *IQR* interquantile range.

### Association between TB at ART initiation and baseline covariates

The final PS model included all 14 baseline covariates, their pairwise interactions with gender and baseline AIDS status (yes or no), and additional quadratic and interaction terms for the continuous variables to improve model fit (Additional file 4). Overall, the PS model predicted baseline TB status well (c statistic = 0.744, p-value = 0.203 for Hosmer and Lemeshow goodness-of-fit test). We were able to match 1,686 of 1,690 baseline TB patients to non-TB patients. PS matching improved the similarity of the distributions of all baseline covariates between TB and non-TB patients and reduced the standardized differences to below 0.1. Table 2 displays the balancing distributions of the baseline covariates for one of the five imputed datasets. Consistent patterns were observed across the imputed datasets. Additional details on specific balance diagnostics of the two continuous covariates are in the appendices (Additional file 5: Figure S2 and Additional file 6: Figure S3).

**Table 2:** **Comparison of baseline and follow**-**up characteristics between HIV**-**infected patients with and without co**-**infection of tuberculosis in propensity score matched pairs with imputed covariates** (**for a single multiple imputation dataset**)

**Covariate**	**TB: yes**	**TB: no**	**Standardized difference**
	**(n = 1686)**	**(n = 1686)**	**(Ratio of variance for continuous covariate)**
**Baseline characterstics**			
***Demographics***			
Age (year)			
Mean (SD)	36.89 (8.77)	36.93 (8.87)	−0.005 (0.978)
Min	14	14	
25th percentile	31	31	
50th percentile	36	36	
75th percentile	42	42	
Max	73	77	
Male: n (%)	668 (39.62)	673 (39.92)	−0.006
***HIV severity***			
CD4 count			
Mean (SD)	143.70 (149.98)	144.34 (139.78)	−0.004 (1.15)
Min	0	0	
25th percentile	45	44	
50th percentile	112	118	
75th percentile	194	196	
Max	1701	1247	
WHO stage: n (%)			
1	43 (2.55)	43 (2.55)	-
2	441 (26.16)	436 (25.86)	
3	996 (59.07)	983 (58.30)	
4	206 (12.22)	224 (13.29)	
AIDS: n (%)	489 (29.00)	479 (28.41)	0.013
***History of drug use *&*illnesses*: *n *(%)**			
Pneumocystis pneumonia	25 (1.48)	33 (1.96)	−0.037
Toxoplasmosis	11 (0.65)	8 (0.47)	0.024
Sexually transmitted infection	340 (20.17)	339 (20.11)	0.001
***Partner information*: *n* (%)**			
Married poly	125 (7.41)	136 (8.07)	−0.024
Partner sero-positive	417 (24.73)	431 (25.56)	−0.019
Sexually active	1221 (72.42)	1223 (72.54)	−0.003
***Other characteristics*: *n* (%)**			
Site			
ENT	374 (22.18)	378 (22.42)	-
GUL	161 (9.55)	176 (10.43)	
JIN	276 (16.37)	271 (16.07)	
MAS	130 (7.71)	125 (7.41)	
MBL	144 (8.54)	160 (9.49)	
MBR	116 (6.88)	121 (7.18)	
MSD	61 (3.62)	62 (3.68)	
MUL	211 (12.51)	203 (12.04)	
SOR	29 (1.72)	18 (1.07)	
TOR	184 (10.91)	172 (10.20)	
Education (Higher institute)	58 (3.44)	59 (3.50)	−0.003
ART start year since 2000			
4	23 (1.36)	15 (0.89)	-
5	452 (26.81)	428 (25.39)	
6	372 (22.06)	360 (21.35)	
7	505 (29.95)	548 (32.50)	
8	330 (19.57)	330 (19.57)	
9	4 (0.24)	5 (0.30)	
**Follow**-**up characteristics**			
Death: n (%)	176 (10.44)	137 (8.13)	0.080
Median (IQR) length of follow-up (days)	629 (205–1036)	659 (286–1023)	-

Note: For continuous variables, mean and standard deviation for each group, standardized mean difference and ratio of variances between TB and no-TB patients are reported. For categorical variables, frequency and percentage of the specified level, and standardized difference between TB and no-TB patients are reported, unless noted otherwise. *MI* multiple imputation, *SD* standard deviation, *WHO* World Health Organization, *HIV* human immunodeficiency virus, *AIDS* acquired immune deficiency syndromes, *ENT* Entebbe, *JIN* Jinja, *MAS* Masaka, *MBL* Mbale, *MBR* Mbarara, *MUL* Mulago, *TOR* Tororo, *GUL* Gulu, *SOR* Soroti, *MSD* Masindi, *IQR* interquantile range.

### Association between TB and all-cause mortality

**Figure 1:**
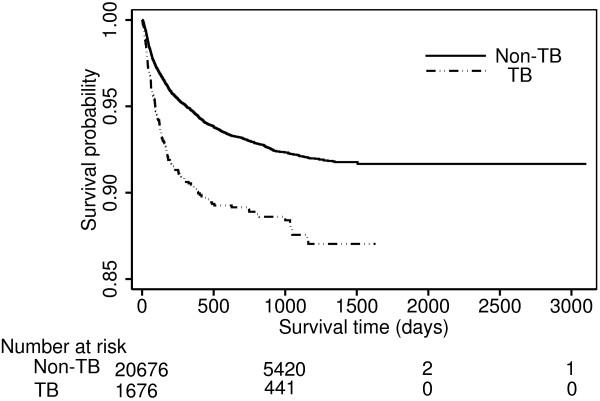
**Kaplan**–**Meier survival curves by baseline TB status in the unmatched study sample.**

**Figure 2:**
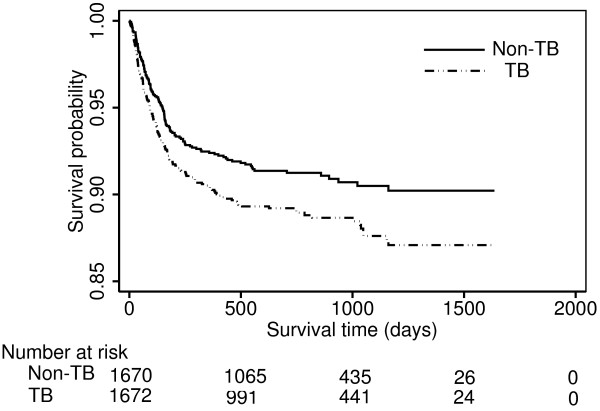
**Kaplan**–**Meier survival curves by baseline TB status in propensity score matched pairs for a single multiple imputation dataset.**

During a median of 21.5 months of follow-up, 1,503 (6.69%) of the 22,477 HIV patients died. The proportions of patients who died with and without TB were 10.47% (median length of follow-up [IQR]: 627.5 days [205–1034]) and 6.38% (median length of follow-up [IQR]: 646 days [306–1045]), respectively, in the original unmatched sample. The proportion of deaths remained higher in the 1,686 PS-matched pairs, with 176 (10.44%; median length of follow-up [IQR]: 629 [205–1036]) and 137 (8.13%; median length of follow-up [IQR]: 659 [286–1023]) deaths in the TB and non-TB groups. An unadjusted Cox model suggested that TB, relative to no TB, increased the hazard of death by 74% (HR = 1.74, 95% CI: 1.49 – 2.04). The HR for all-cause mortality comparing TB to non-TB patients on the 1,686 PS-matched pairs (HR = 1.37, 95% CI: 1.08 – 1.75) was less substantial, indicating TB was associated with a 37% increase in the hazard of death over the course of the study after controlling for potential baseline confounding variables (Table 3). The PS-matched estimate was less precise than the crude due to a decrease of sample size. Kaplan-Meier survival curves for the original sample and PS-matched pairs are displayed in Figures 1 and 2. No violation of the PH assumption was suggested by log-log survival curves.

**Table 3:** **Effect of tuberculosis** (**TB**) **at the initiation of antiretroviral therapy** (**ART**) **on overall survival**

**Method**	**HR**	**95% CI**	**P**-**value**	CI width ratio*
***Primary analysis***				
Matching on PS	1.37	(1.08, 1.75)	0.011	1
***Additional analyses***				
Unadjusted Cox regression	1.74	(1.49, 2.04)	<0.001	0.82
Stratified on PS	1.36	(1.15, 1.60)	<0.001	0.66
PS as covariate (linear, quadratic and cubic terms)	1.34	(1.14, 1.58)	<0.001	0.65
Conventional adjusted Cox regression	1.40	(1.19, 1.65)	<0.001	0.69

Results for the propensity score (PS) or covariate-adjusted Cox regression models were aggregated across five multiple imputation datasets using Rubin’s rule.

*HR* hazard ratio, *CI* confidence interval, *PS* propensity score. * Reference = width of 95% CI for PS-matched estimate.

### Additional analyses

Stratifying on PS quintiles and adjusting for PS as a covariate in Cox regression models yielded similar TB effects on survival (PS-stratified HR = 1.36, 95% CI: 1.15-1.60; PS-adjusted HR = 1.34, 95% CI: 1.14 -1.58) (Table 3). Adjusting for multiple baseline covariates simultaneously in the conventional not PS-based Cox model did not alter the association substantially (HR = 1.40, 95% CI: 1.19-1.65). Ratios of the widths of the 95% CIs are presented in Table 3. HR estimates using the alternative adjustment methods had greater precision relative to the result of the PS-matched analysis, as the latter only utilized data from the matched pairs, a subset of the study sample. The trade-off between accuracy and precision of the PS methods has been noted in previous studies [40,42].

## Discussion

This is, to our knowledge, the largest cohort of HIV-infected patients receiving ART in a single African country that has evaluated mortality outcomes in co-infected patients. Descriptive analysis showed that TB patients, compared to non-TB patients, were more likely to be immunosuppressed, have a history of opportunistic infections, and live in particular geographical regions of Uganda. The results of Cox PH analysis using PS matching for confounding control showed that having pulmonary TB disease at the initiation of ART led to a 37% increase in the hazard of all-cause mortality during the follow-up period between 2000 and 2009. Additional analyses using two other PS methods and the conventional (not PS-based) multivariable Cox regression model showed similar results. These analyses may be viewed as sensitivity analyses because different methods use different assumptions and the results seem to remain robust to different ways of adjusting for baseline confounding.

The prevalence of active pulmonary TB at the initiation of ART of 7.52% in the study is consistent with results from a smaller TASO cohort reported from Tororo, Uganda [15]. This indicates that TB is less prevalent in Uganda compared with other African countries, particularly Southern Africa [16]. An open cohort of 7,512 patients receiving ART in South Africa suggested that 15.9% of HIV patients were treated for pulmonary TB at ART initiation between 2004 and 2007. Our finding of a 37% increase in hazard of overall mortality associated with having active TB at ART initiation after controlling for potential baseline confounding is both statistically significant and clinically relevant. This result is different from Westreich et al.’s findings on 7,512 HIV patients in South Africa, indicating pulmonary TB treatment at ART initiation was uncorrelated to the overall survival (HR = 1.06, 95% CI: 0.75-1.49) after adjusting for multiple confounders [16]. While all TB patients in the South Africa study received ART soon after initiation of TB treatment if TB treatment was well tolerated, the current study did not have reliable data on TB treatment at the initiation of ART. Instead the effect of baseline active pulmonary TB disease on mortality was evaluated and active TB patients might or might not have received TB treatment during the course of the study. The difference in baseline exposure between the two studies is likely to be the most important factor explaining the different results and suggests a benefit of starting TB treatment at ART initiation to reduce overall mortality in a co-infected population.

Guidelines on the treatment of TB co-infection with HIV have changed dramatically over the last few years and may not reflect within-country variability. For example, early guidelines feared drug interactions between TB medication and ART or negative impact on adherence of ART or TB due to pill burden and suggested early treatment for TB disease followed by stabilization with ART. However, more recent evidence from randomized trials [4-6] suggests that starting ART within two weeks of TB treatment to improve survival appears to be well tolerated, and has persuaded guideline makers to recommend co-administration of treatment. In addition, recent data from South Africa found that although IRIS events were associated with slightly lower adherence rates, overall adherence to ART remained high in this co-infected population. The data suggested that concerns about IRIS or drug-drug interactions should not deter clinicians from early ART initiation, but patients developing IRIS events should be monitored closely and potentially targeted for interventions to increase adherence [52]. Other developments are related to the specific setting. Uganda, for example, has a lower national prevalence of co-infection than Southern Africa and has not reported any cases of extensively drug resistant (XDR) TB infections. South Africa, on the other hand, has had an epidemic of XDR co-infections that has resulted in quarantine of some patients and recommendations about interacting with other individuals [53]. National guidelines from Uganda do not match those of South Africa, as discussed earlier.

Limitations of the study emerge from three key sources. First, propensity score methods cannot balance unmeasured confounders in estimating a causal relationship, which is an issue faced by all observational cohorts. Our results may be biased if some unobserved prognostic factors were unbalanced between TB and non-TB groups. While some studies examined the impact of body mass index and anemia on clinical outcomes in co-infected patients [16], such baseline information was unavailable in the current study. More resources need to be dedicated to improve the completeness and accuracy of data collection in HIV patients in underdeveloped regions. Although statistical tools instrumental variable analysis, have been developed to control for bias due to unmeasured confounders [54], strong assumptions are required to ensure estimation accuracy, and such assumptions can be difficult to verify using empirical data. Second, the multiple imputation method employed to impute the missing values for baseline covariates requires missing at random (MAR) assumption (i.e. missingness can be explained by differences in the observed data). Although the current imputation procedure included a number of baseline characteristics and follow-up variables, we could not rule out the existence of additional variables that were highly associated with missingness. The MAR assumption is unverifiable using data collected within a study. Third, our results are predominantly based on adult, HIV-infected, African patients who initiated ART in Uganda, during 2004 to 2009, hence limiting their generalizability. The effect of active pulmonary TB disease on HIV patients in other age or ethnic groups (for instance, children and adolescents) and from different geographic regions should be further investigated.

Our study shows a 37% increase in the hazard of death among co-infected patients compared with those only infected with HIV. We used pulmonary TB disease as documented through sputum and radiologic confirmation for the diagnosis of TB disease as TASO, along with most AIDS service organizations in Africa, lacked the infrastructure to consistently diagnose extra-pulmonary TB disease. Also, we recognize that sensitivity of sputum smear for diagnosis of pulmonary TB in HIV-infected patients was poor (less than 50%) and thus we followed up patients with suspect TB disease with radiologic examinations regardless of sputum results. Because TB is associated with mortality (as observed in this study), misclassifying actual active TB patients into the non-TB group would attenuate the magnitude of the TB effect on death; as a result the apparent estimates of TB effects on death would be less substantial than the actual effects.

## Conclusions

In summary, our current study suggests a moderate increase in the hazard of death associated with having active TB disease at the initiation of ART in HIV-infected patients receiving ART in Uganda. Our finding is complementary to results of the recent SAPIT, [4] CAMELIA, [5] and STRIDE trials [6] that demonstrate improved survival when ART is initiated during TB therapy. The results also validate the WHO guidelines that urge a more aggressive approach to management of both TB and HIV [55].

## Abbreviations

TB: Tuberculosis; ART: Antiretroviral therapy; HIV: Human immunodeficiency virus; AIDS: Acquired immune deficiency syndromes; WHO: World Health Organization; TASO: AIDS Support Organization; IRIS: Immune reconstitution inflammatory syndrome; PCP: Pneumocystis pneumonia; PS: Propensity score; HR: Hazard ratio; MI: Multiple imputation; MAR: Missing at random; SD: Standard deviation; CI: Confidence interval; ENT: Entebbe; JIN: Jinja; MAS: Masaka; MBL: Mbale; MBR: Mbarara; MUL: Mulago; TOR: Tororo; GUL: Gulu; SOR: Soroti; MSD: Masindi; IQR: Interquantile range.

## Competing interests

The authors declare that they have no competing interest.

## Authors’ contributions

RC participated in the conception and design of the study, data cleaning and analysis, results interpretation, and drafting and revision of the manuscript. EM participated in the conception and design of the study, data collection, results interpretation, and revision of the manuscript. JB and EP participated in review of statistical methods, results interpretation, and revision of the manuscript. CN participated in data collection from TASO clinics and review of drafts. JBN and PJD participated in results interpretation and revision of the manuscript. LT participated in the conception and design of the study, results interpretation, and revision of the manuscript. All authors read and approved the final manuscript.

## Supplementary Material

Additional file 1 Coding of baseline covariates and post-ART variables. Click here for file

Additional file 2: Figure S1 Study flowchart. Click here for file

Additional file 3 Summary statistics of the four baseline covariates with missing values in the original and imputed datasets. Click here for file

Additional file 4 Results of the final propensity score (PS) model. Click here for file

Additional file 5: Figure S2 Distribution of age (in years) at ART initiation in TB and no-TB group in the original sample and PS-matched pairs for a single MI dataset. From top to bottom: quantile-quantile plots, empirical cumulative distribution functions (circles represent TB and triangles represent no-TB), nonparametric density curves (solid line represents TB and dashed line represents no-TB), and side-by-side boxplots. Click here for file

Additional file 6: Figure S3 Distribution of CD4 cell counts at ART initiation in TB and no-TB group in the original sample and PS matched pairs for a single MI dataset. From top to bottom: quantile-quantile plots, empirical cumulative distribution functions (circles represent TB and triangles represent no-TB), nonparametric density curves (solid line represents TB and dashed line represents no-TB), and side-by-side boxplots. Click here for file
