# Supplementary material for: Impact of tuberculosis on mortality among HIV-infected patients receiving antiretroviral therapy in Uganda: a prospective cohort analysis
Source: AIDS Res Ther. 2013 Jul 13;10:19. doi: 10.1186/1742-6405-10-19 (PMC3716897; doi:10.1186/1742-6405-10-19)
Supplement: Additional file 1 — Coding of baseline covariates and post-ART variables. [file 1742-6405-10-19-S1.docx]

**Coding for the 14 baseline covariates measured at the initiation of ART and variables measure post ART. Post ART variables were excluded from the PS model yet used for imputing the four missing baseline covariates.**

The 14 baseline covariates: gender (male or female), age (continuous), CD4 count (continuous), World Health Organization (WHO) clinical disease stage of HIV/AIDS (4 categories: stages 1 - 4, reference = stage 1) , presence of AIDS-defining illness (yes or no), TASO service centre (10 centres, reference = Entebbe), calendar year of ART initiation (6 categories: 2000, 2004, 2005, 2006, 2007, 2008, 2009; reference = 2000), education (higher institute education or not), marital status (married mono or others), partner sero-status (positive or negative), sexual activity (sexually active or not), sexually transmitted infection (yes or no), history of pneumocystis pneumonia (yes or no), and toxoplasmosis (yes or no).

Post-ART variables: death (yes or no), regimen switch (yes or no), patient adherence (yes or no), and AIDS status post-ART initiation (yes or no).
