# Supplementary figures and images for: Impact of tuberculosis on mortality among HIV-infected patients receiving antiretroviral therapy in Uganda: a prospective cohort analysis
Source: AIDS Res Ther. 2013 Jul 13;10:19. doi: 10.1186/1742-6405-10-19 (PMC3716897; doi:10.1186/1742-6405-10-19)

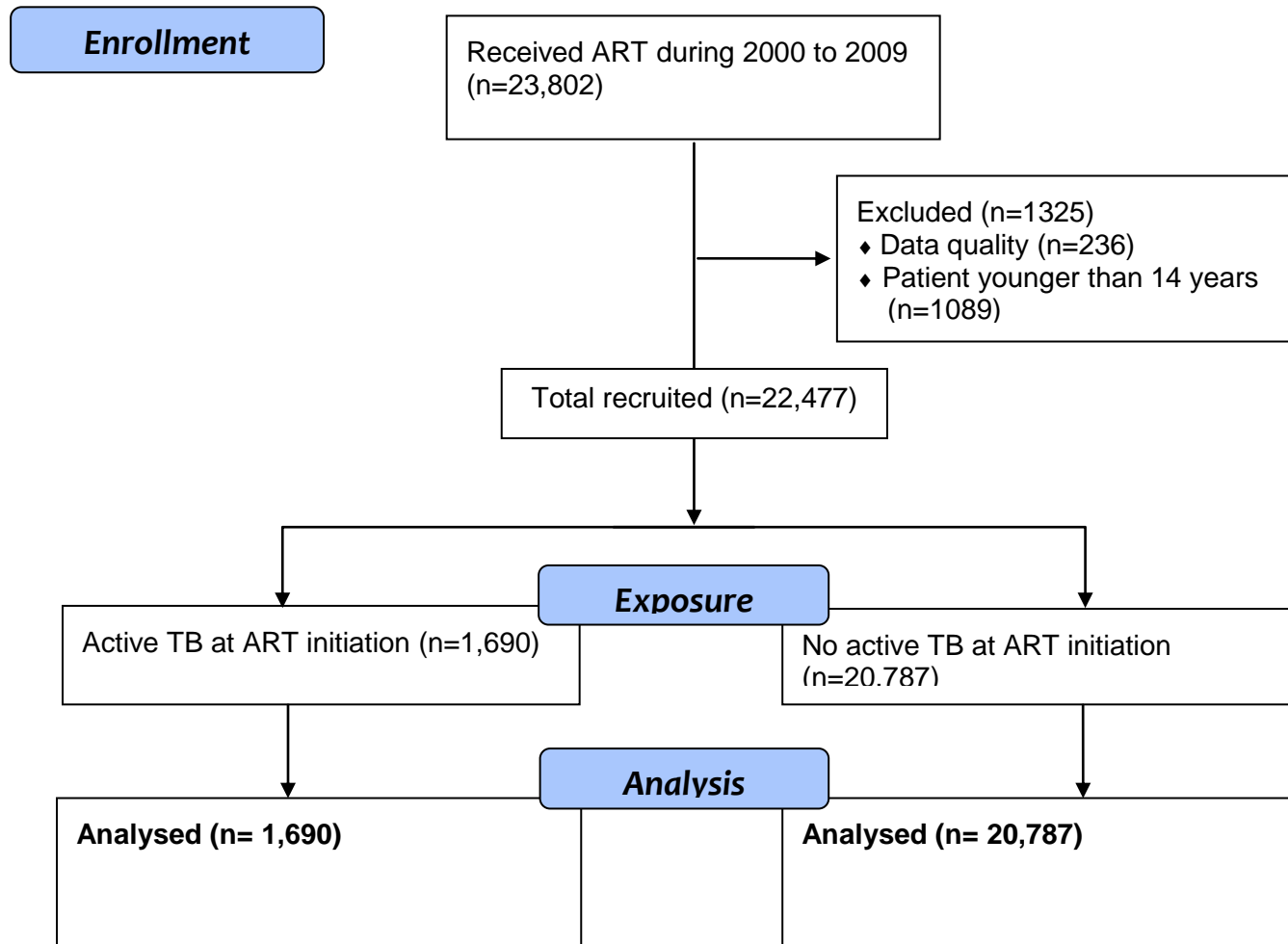

Supplement: Additional file 2: Figure S1 — Study flowchart. [file 1742-6405-10-19-S2.pdf]

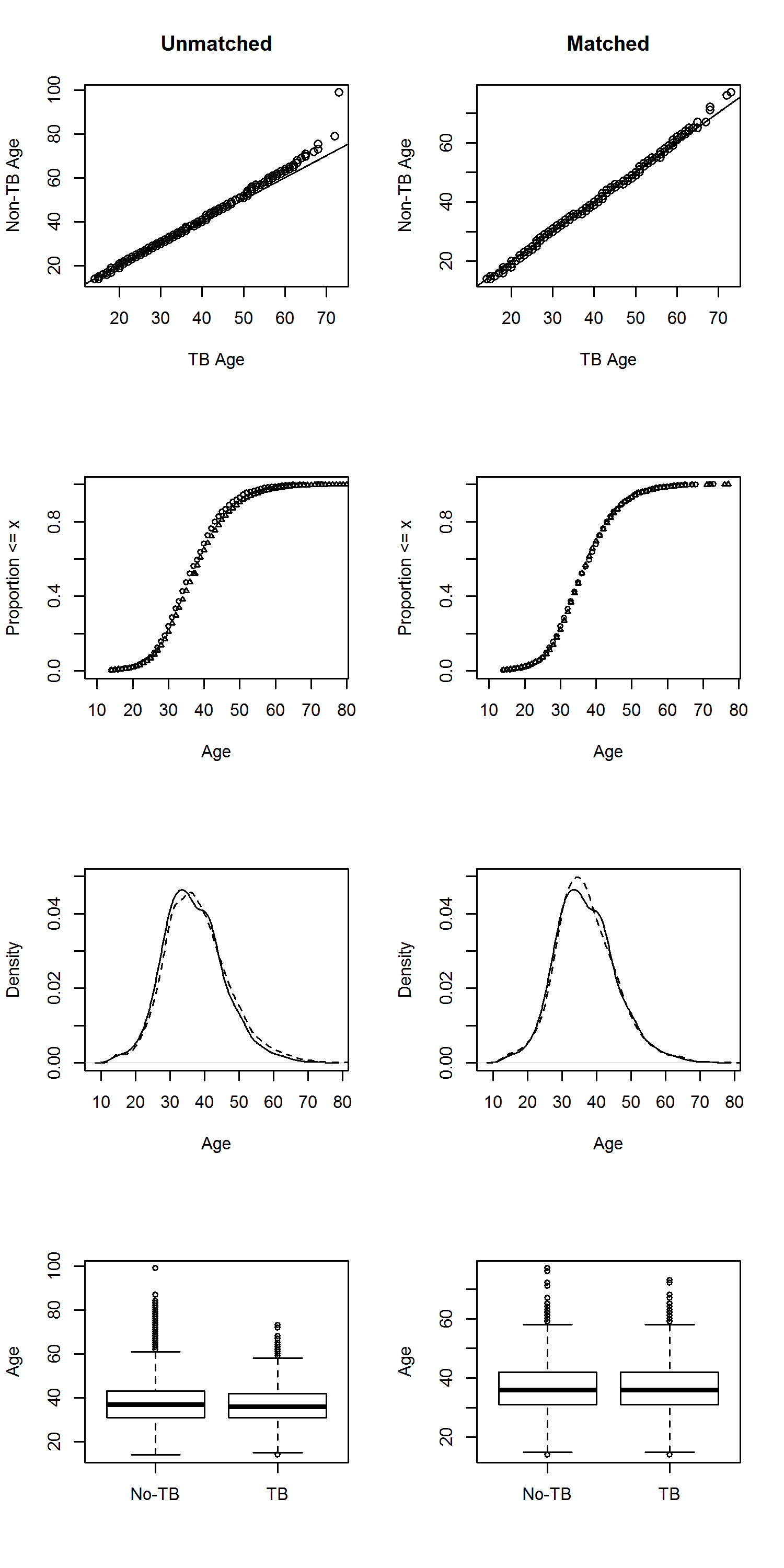

Supplement: Additional file 5: Figure S2 — Distribution of age (in years) at ART initiation in TB and no-TB group in the original sample and PS-matched pairs for a single MI dataset. From top to bottom: quantile-quantile plots, empirical cumulative distribution functions (circles represent TB and triangles represent no-TB), nonparametric density curves (solid line represents TB and dashed line represents no-TB), and side-by-side boxplots. [file 1742-6405-10-19-S5.tiff]

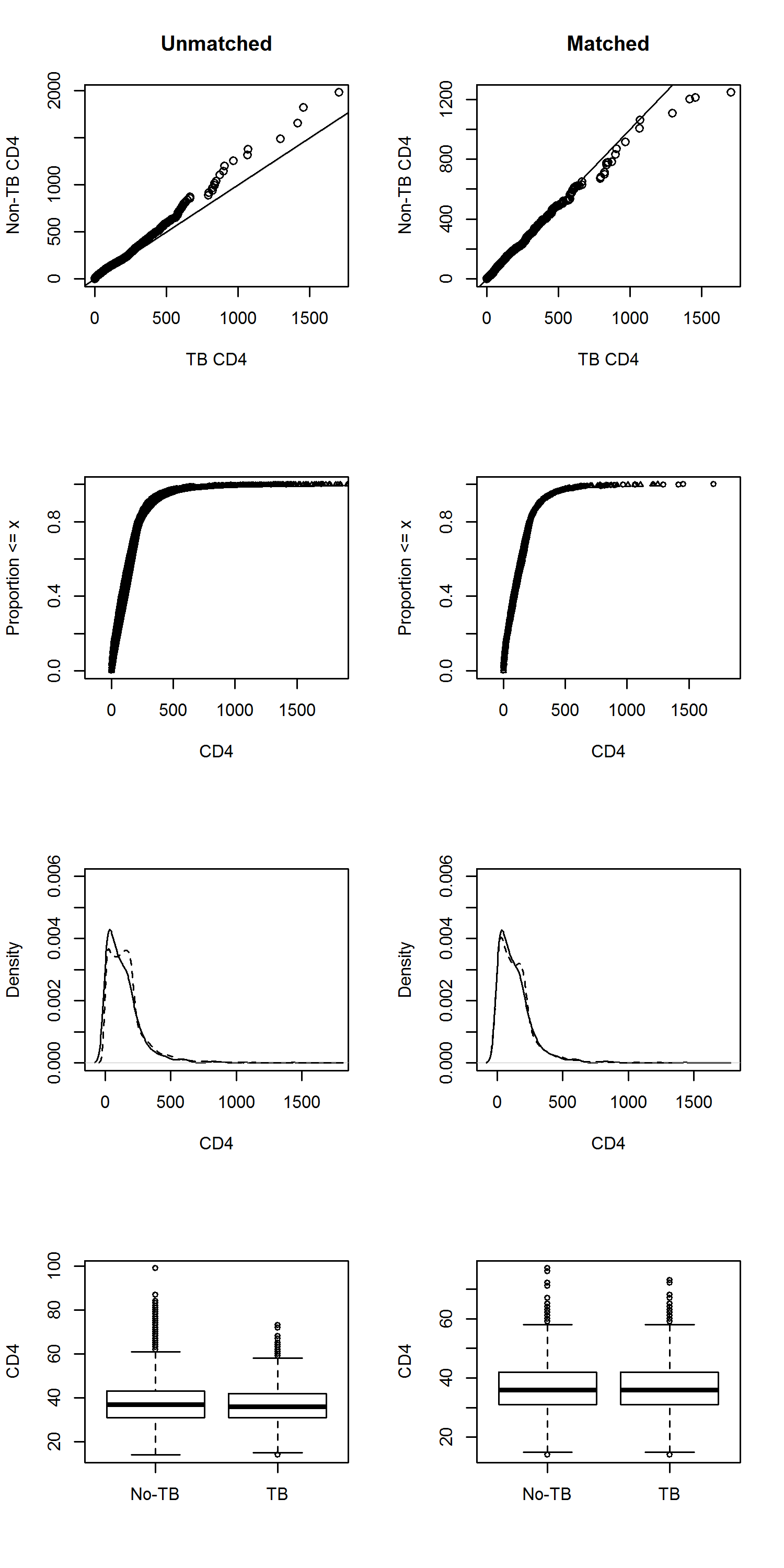

Supplement: Additional file 6: Figure S3 — Distribution of CD4 cell counts at ART initiation in TB and no-TB group in the original sample and PS matched pairs for a single MI dataset. From top to bottom: quantile-quantile plots, empirical cumulative distribution functions (circles represent TB and triangles represent no-TB), nonparametric density curves (solid line represents TB and dashed line represents no-TB), and side-by-side boxplots. [file 1742-6405-10-19-S6.tiff]
