# Supplementary material for: Impact of tuberculosis on mortality among HIV-infected patients receiving antiretroviral therapy in Uganda: a prospective cohort analysis
Source: AIDS Res Ther. 2013 Jul 13;10:19. doi: 10.1186/1742-6405-10-19 (PMC3716897; doi:10.1186/1742-6405-10-19)
Supplement: Additional file 3 — Summary statistics of the four baseline covariates with missing values in the original and imputed datasets. [file 1742-6405-10-19-S3.docx]

**Summary statistics of the four baseline covariates with missing values in the original and imputed datasets**

Summary statistics of age, CD4, WHO disease stage of HIV/AIDS and sexual activity in the original study sample (without multiple imputation)

| **Variable** | **N** | **Mean** | **SD** | **25^th^ percentile** | **Median** | **75^th^ percentile** |
| --- | --- | --- | --- | --- | --- | --- |
| Age  CD4  Disease stage  Sexual activity | 22460  18592  14741  18590 | 37.85  163.79  2.48  0.73 | 9.44  147.37  0.69  0.44 | 31  70  2  0 | 37  142  2  1 | 43  206  3  1 |

*SD: standard deviation*

*N: number of patients with complete data on the variable*

Summary statistics of age, CD4, WHO stage of HIV/AIDS and sexual activity in the five imputated datasets

| **Imputation Number** | **N** | **Variable** | **Mean** | **SD** | **25^th^ percentile** | **Median** | **75^th^ percentile** |
| --- | --- | --- | --- | --- | --- | --- | --- |
| 1 | 22477 | Age CD4 Disease stage Sexual activity | 37.85 168.76 2.48  0.73 | 9.44 170.84 0.71  0.44 | 31 64 2  0 | 37 137 2.00  1 | 43 207 3  1 |
| 2 | 22477 | Age CD4 Disease stage Sexual activity | 37.85 168.01 2.48 0.74 | 9.44 168.06 0.71 0.44 | 31 64 2 0 | 37 136 2 1 | 43 207 3 1 |

| 3 | 22477 | Age CD4 Disease stage Sexual activity | 37.85 166.87 2.47 0.73 | 9.44 165.31 0.71 0.44 | 31 64 2 0 | 37 136 2 1 | 43 206 3 1 |
| --- | --- | --- | --- | --- | --- | --- | --- |
| 4 | 22477 | Age CD4 Disease stage Sexual activity | 37.85 167.60 2.48 0.74 | 9.44 167.56 0.71 0.44 | 31 64 2 0 | 37 137 2 1 | 43 207 3 1 |
| 5 | 22477 | Age CD4 Disease stage Sexual activity | 37.85 168.01 2.48 0.74 | 9.44 169.67 0.70 0.44 | 31 64 2 0 | 37 136 2 1 | 43 207 3 1 |

*SD: standard deviation*

*N: number of subjects with complete or imputed data for the variable*
