# Supplementary material for: Impact of tuberculosis on mortality among HIV-infected patients receiving antiretroviral therapy in Uganda: a prospective cohort analysis
Source: AIDS Res Ther. 2013 Jul 13;10:19. doi: 10.1186/1742-6405-10-19 (PMC3716897; doi:10.1186/1742-6405-10-19)
Supplement: Additional file 4 — Results of the final propensity score (PS) model. [file 1742-6405-10-19-S4.docx]

**Results of the final propensity score (PS) model**

Table S1 Results of the final PS model (logistic regression)

| **Parameter** | **Log OR** | **SE** | **P-value** |
| --- | --- | --- | --- |
| Intercept | -10.15 | 203.80 | 0.960 |
| Log CD4 | -0.24 | 0.16 | 0.139 |
| Age | 0.05 | 0.03 | 0.027 |
| Start year 2009 | 7.82 | 203.80 | 0.969 |
| Start year 2008 | 6.99 | 203.80 | 0.973 |
| Start year 2007 | 7.06 | 203.80 | 0.972 |
| Start year 2006 | 7.28 | 203.80 | 0.971 |
| Start year 2005 | 7.27 | 203.80 | 0.971 |
| Start year 2004 | 5.91 | 203.80 | 0.977 |
| Male | -1.83 | 288.20 | 0.995 |
| Site TOR | -0.10 | 0.14 | 0.477 |
| Site SOR | -1.91 | 0.31 | <0.001 |
| Site MUL | -0.46 | 0.14 | 0.001 |
| Site MSD | -1.01 | 0.22 | <0.001 |
| Site MBR | -1.32 | 0.17 | <0.001 |
| Site MBL | -0.85 | 0.16 | <0.001 |
| Site MAS | -1.21 | 0.16 | <0.001 |
| Site JIN | -0.32 | 0.13 | 0.017 |
| Site GUL | -0.46 | 0.15 | 0.002 |
| AIDS | 1.21 | 1.03 | 0.239 |
| No higher institute education | -0.08 | 0.23 | 0.745 |
| Married mono | 0.08 | 0.14 | 0.586 |
| Partner sero positive | -0.16 | 0.10 | 0.118 |
| Sexually transmitted infection | -0.03 | 0.09 | 0.708 |
| Pneumocystis pneumonia | 1.08 | 0.35 | 0.002 |
| Toxoplasmosis | 0.44 | 0.56 | 0.430 |
| Sexually active | -0.18 | 0.09 | 0.041 |
| WHO stage 4 | 1.44 | 0.99 | 0.148 |
| WHO stage 3 | 1.79 | 0.93 | 0.054 |
| WHO stage 2 | 0.40 | 0.96 | 0.673 |
| Log CD4*Male | 0.02 | 0.04 | 0.578 |
| Age*Male | -0.01 | 0.01 | 0.250 |
| Start year 2009*Male | 4.34 | 288.20 | 0.988 |
| Start year 2008*Male | 3.22 | 288.20 | 0.991 |
| Start year 2007*Male | 3.07 | 288.20 | 0.991 |
| Start year 2006*Male | 3.06 | 288.20 | 0.991 |
| Start year 2005*Male | 3.01 | 288.20 | 0.992 |
| Start year 2004*Male | 3.02 | 288.20 | 0.992 |
| Site TOR*Male | -0.52 | 0.21 | 0.012 |
| Site SOR*Male | 0.08 | 0.40 | 0.837 |
| Site MUL*Male | -0.63 | 0.20 | 0.002 |
| Site MSD*Male | -0.43 | 0.30 | 0.150 |
| Site MBR*Male | -0.17 | 0.23 | 0.469 |
| Site MBL*Male | -0.19 | 0.22 | 0.395 |
| Site MAS*Male | -0.35 | 0.23 | 0.119 |
| Site JIN*Male | -0.45 | 0.19 | 0.016 |
| Site GUL*Male | -0.47 | 0.22 | 0.031 |
| AIDS*Male | 0.11 | 0.14 | 0.421 |
| No higher institute education * Male | -0.02 | 0.30 | 0.953 |
| Married mono * Male | 0.10 | 0.21 | 0.613 |
| Partner sero positive * Male | 0.03 | 0.13 | 0.831 |
| Sexually transmitted infection * Male | 0.01 | 0.16 | 0.948 |
| Pneumocystis pneumonia * Male | 0.40 | 0.49 | 0.417 |
| Toxoplasmosis*Male | -0.56 | 0.76 | 0.458 |
| Sexually active*Male | 0.01 | 0.14 | 0.937 |
| WHO stage 4*Male | -0.53 | 0.37 | 0.152 |
| WHO stage 3*Male | -0.37 | 0.34 | 0.272 |
| WHO stage 2*Male | -0.52 | 0.34 | 0.132 |
| (Log CD4)^2^ | -0.001 | 0.01 | 0.846 |
| (Age)^2^ | -0.0005 | 0.0002 | 0.033 |
| WHO stage 4 * Log CD4 | 0.17 | 0.16 | 0.290 |
| WHO stage 3* Log CD4 | 0.19 | 0.15 | 0.223 |
| WHO stage 2* Log CD4 | 0.19 | 0.16 | 0.223 |
| WHO stage 4 * Age | -0.02 | 0.02 | 0.198 |
| WHO stage 3 * Age | -0.03 | 0.02 | 0.057 |
| WHO stage 2 * Age | -0.03 | 0.02 | 0.084 |
| Log CD4 * AIDS | -0.01 | 0.04 | 0.874 |
| Age * AIDS | 0.002 | 0.01 | 0.827 |
| Start year 2009 * AIDS | -1.73 | 1.71 | 0.311 |
| Start year 2008 * AIDS | -0.66 | 0.79 | 0.398 |
| Start year 2007 * AIDS | -0.70 | 0.78 | 0.370 |
| Start year 2006 * AIDS | -0.93 | 0.79 | 0.237 |
| Start year 2005 * AIDS | -0.58 | 0.78 | 0.459 |
| Site TOR * AIDS | -0.64 | 0.25 | 0.009 |
| Site SOR * AIDS | -0.19 | 0.46 | 0.675 |
| Site MUL * AIDS | -0.08 | 0.20 | 0.682 |
| Site MSD * AIDS | -0.45 | 0.33 | 0.174 |
| Site MBR * AIDS | -0.09 | 0.24 | 0.722 |
| Site MBL * AIDS | -0.47 | 0.24 | 0.054 |
| Site MAS * AIDS | -0.47 | 0.25 | 0.064 |
| Site JIN * AIDS | -0.40 | 0.20 | 0.045 |
| Site GUL * AIDS | -0.22 | 0.27 | 0.416 |
| No higher institute education * AIDS | 0.38 | 0.35 | 0.281 |
| Married mono * AIDS | -0.04 | 0.23 | 0.872 |
| Partner sero positive * AIDS | -0.14 | 0.15 | 0.354 |
| Sexually transmitted infection * AIDS | -0.03 | 0.14 | 0.845 |
| Pneumocystis pneumonia * AIDS | 0.08 | 0.47 | 0.868 |
| Toxoplasmosis * AIDS | -0.55 | 0.70 | 0.437 |
| Sexually active * AIDS | 0.26 | 0.14 | 0.072 |
| WHO stage 4 * AIDS | -0.56 | 0.42 | 0.184 |
| WHO stage 3 * AIDS | -0.71 | 0.40 | 0.077 |
| WHO stage 2 * AIDS | -0.21 | 0.41 | 0.605 |

*Log OR: log odds ratio, SE: standard error, p-value: Wald Chi-squared p-value, WHO: World Health Organization, HIV: human immunodeficiency virus, AIDS: acquired immune deficiency syndromes, ENT: Entebbe, JIN: Jinja, MAS: Masaka, MBL: Mbale, MBR: Mbarara, MUL: Mulago, TOR: Tororo, GUL: Gulu, SOR: Soroti, MSD: Masindi*
